# TRPC1/TRPC3 channels mediate lysophosphatidylcholine-induced apoptosis in cultured human coronary artery smooth muscles cells

**DOI:** 10.18632/oncotarget.10853

**Published:** 2016-07-26

**Authors:** Yuan Wang, Yan Wang, Gui-Rong Li

**Affiliations:** ^1^ Xiamen Cardiovascular Hospital, Medical School of Xiamen University, Xiamen, Fujian, China; ^2^ Department of Medicine, Li Ka Shing Faculty of Medicine, University of Hong Kong, Pokfulam, Hong Kong, China

**Keywords:** lysophosphatidylcholine, cell viability, apoptosis, transient receptor potential channels, Pathology Section

## Abstract

The earlier study showed that lysophosphatidylcholine (lysoPC) induced apoptosis in human coronary artery smooth muscle cells (SMCs); however, the related molecular mechanisms are not fully understood. The present study investigated how lysoPC induces apoptosis in cultured human coronary artery SMCs using cell viability assay, flow cytometry, confocal microscopy, and molecular biological approaches. We found that lysoPC reduced cell viability in human coronary artery SMCs by eliciting a remarkable Ca^2+^ influx. The effect was antagonized by La^3+^, SKF-96365, or Pyr3 as well as by silencing TRPC1 or TRPC3. Co-immunoprecipitation revealed that TRPC1 and TRPC3 had protein-protein interaction. Silencing TRPC1 or TRPC3 countered the lysoPC-induced increase of Ca^2+^ influx and apoptosis, and the pro-apoptotic proteins Bax and cleaved caspase-3 and decrease of the anti-apoptotic protein Bcl-2 and the survival kinase pAkt. These results demonstrate the novel information that TRPC1/TRPC3 channels mediate lysoPC-induced Ca^2+^ influx and apoptosis via activating the pro-apoptotic proteins Bax and cleaved caspase-3 and inhibiting the anti-apoptotic protein Bcl-2 and the survival kinase pAkt in human coronary artery SMCs, which implies that TRPC1/TRC3 channels may be the therapeutic target of lysoPC-induced disorders such as atherosclerosis.

## INTRODUCTION

Oxidized low-density lipoprotein (ox-LDL) is a well-known culprit of atherogenesis, which causes a series of aberrant initial changes in vascular smooth muscle cells (SMCs) including lipid homeostasis and deposition, dysfunction of Ca^2+^ homeostasis, and apoptosis [1, 2]. Lysophosphatidylcholine (lysoPC) is a major component of ox-LDL [3, 4], and induces apoptosis of vascular endothelial cells and SMCs [5–7]. However, the related molecular mechanisms are not fully understood.

Ca^2+^ is a well-known second messenger that regulates a wide range of cell functions including excitation-contraction coupling, excitation-secretion coupling, gene transcription, cell growth, differentiation, apoptosis, membrane fusion, and ion channel activation [8–11]. Previous studies reported that lysoPC induced an sustained increase in intracellular free Ca^2+^ (Ca^2+^_i_) in human [12] and rabbit [13] coronary artery SMCs and in human umbilical vein endothelial cells [14] and in cultured human corporal SMCs [15]. The participation of transient receptor potential (TRP) channels was proposed in these reports. However, TRP channels contains 6 families (TRPC, TRPV, TRPM, TRPP, TRPML, and TRPA) with different isoforms in each subfamily [16]. It is unknown which TRP channels mediate the nonselective cation current and/or Ca^2+^ entry, and lysoPC-induced apoptosis. The present study was therefore designed to determine the potential molecular pathway that mediates the lysoPC-induced Ca^2+^ influx and/or apoptosis in human coronary artery SMCs. Our results revealed that TRPC1/TRPC3 channels were involved in mediating lysoPC-induced Ca^2+^ influx and apoptosis by increasing the pro-apoptosis kinases Bax and cleaved caspase-3 and inhibiting the anti-apoptosis kinase Bcl-2 and the surviving kinase p-Akt in human coronary artery SMCs.

## RESULTS

### Effect of lysoPC on cell viability

**Figure 1:**
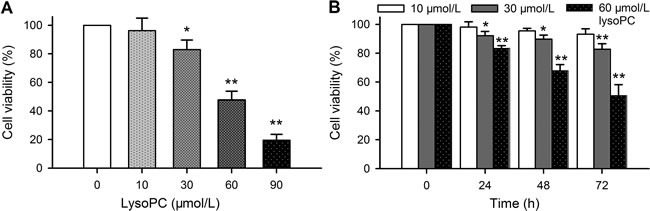
Effect of lysoPC on cell viability **A.** Cell viability was determined with MTT assay in human coronary artery SMCs incubated with 10, 30, 60, and 90 μmol/L lysoPC for 72 h. Data are mean ± SEM (*P < 0.05, **P < 0.01 *vs*. control, 0 μmol/L lysoPC). **B.** Cell viability was determined in human coronary artery SMCs incubated with 10, 30, and 60 μmol/L lysoPC for 24-72 h. Data are mean ± SEM (*n* = 3 individual experiments, *P < 0.05, **P < 0.01 *vs*. 0 day).

The previous studies reported that lysoPC decreased cell viability in cultured rat aortic artery SMCs [17]. The effect of lysoPC on cell viability was confirmed with MTT assay in human coronary artery SMCs. Figure 1A illustrates that a 72 h incubation of lysoPC (10-90 μmol/L) induced a reduction of cell viability in a concentration-dependent manner, and significant decrease of viability was observed at concentrations greater than 30 μmol/L lysoPC. The time-dependent inhibition of cell viability was determined with 10, 30 and 60 μmol/L lysoPC (Figure 1B). Significant reduction of cell viability was observed with 30 and 60 μmol/L at 24-72 h. LysoPC at 60 μmol/L demonstrated a remarkable inhibitory effect on cell viability.

### Apoptosis induced by lysoPC

**Figure 2:**
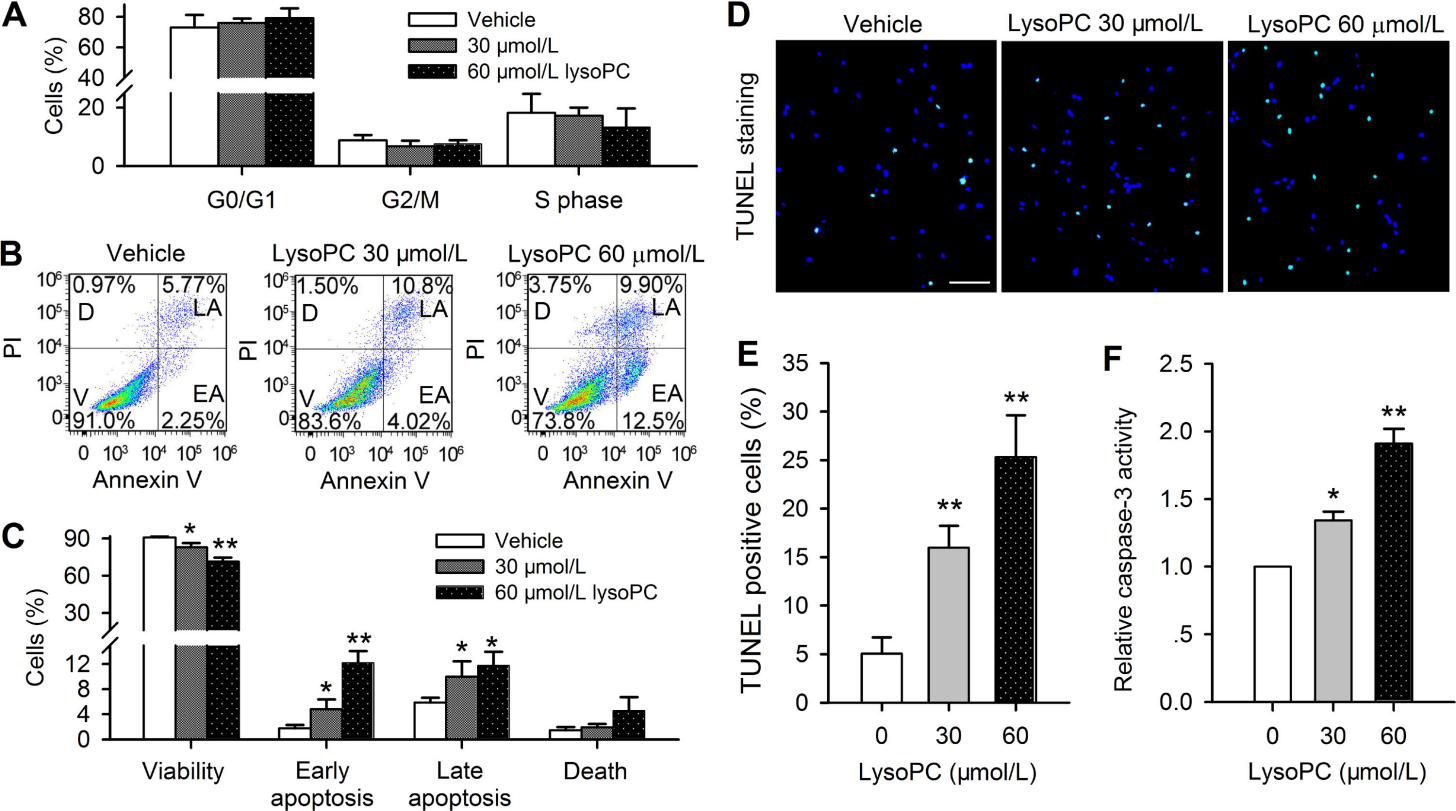
Flow cytometry analysis in cell treated with lysoPC **A.** Cell cycling progression was not affected by 30 or 60 μmol/L lysoPC (24 h) in human coronary artery SMCs. **B.** Flow cytometry analysis was made in cells treated with 30 or 60 μmol/L (24 h) and co-stained with PI and Annexin V-FITC (V, viability; D, death; LA, late apoptosis; EA, early apoptosis) **C.** Percentage of cells that show viability, early apoptosis, late apoptosis and death after treatment with 30 or 60 μmol/L lysoPC (*n* = 3, *P < 0.05, **P < 0.01 *vs*. vehicle control). **D.** Representative microphotographs of TUNEL staining in human coronary artery SMCs treated with 30 or 60 μmol/L lysoPC (24 h). TUNEL labelling is stained in green and nuclei are labelled by DAPI staining in blue. Scale bar = 50 μm. **E.** The percentage of TUNEL-positive cells in total cells. The DAPI staining cells were countered from five randomly picked regions (**P < 0.01 *vs*. vehicle control). **F.** Detection of the caspase-3 activity in human coronary artery SMCs treated with 30 or 60 μmol/L lysoPC for 24 h. (*n* = 3 individual experiments, *P < 0.05, **P < 0.01 *vs*. 0, i.e. vehicle control).

Whether the reduced cell viability is a result of inhibition of cell cycling progression was analyzed with flow cytometry. Figure 2A shows the percent values of cells at different cycling progression phases. No significant change in cell cycling progression phases was observed in cells treated with 30 or 60 μmol/L lysoPC for 24 h, suggesting that the decreased viability by lysoPC is not related to cell cycling arrest in human coronary artery SMCs.

To determine whether the reduced cell viability is related to apoptosis, flow cytometry analysis was further performed in human coronary artery SMCs treated with 30 or 60 μmol/L lysoPC (24 h) and stained with Annexin V-FITC, showing reduced viability and increased apoptosis (Figure 2B). Figure 2C shows the mean percent values of cell viability, early apoptosis, late apoptosis and death. The cell viability was reduced by 30 and 60 μmol/L lysoPC (*n* = 3 individual experiments, *P* < 0.05 or *P* < 0.01 *vs*. control) similar to MTT assay (Figure 1). The percent values of cell populations at early apoptosis and late apoptosis were increased from 1.8 ± 0.5% and 5.9 ± 0.8% of control to 4.8 ± 0.8% and 10.0 ± 1.8% by 30 μmol/L lysoPC (*n* = 3, *P* < 0.05 *vs*. vehicle control), to 12.2 1.8% and 11.8 ± 2.0% by 60 μmol/L lysoPC (*n* = 3, *P* < 0.01 *vs*. vehicle control). The dead cells were not significantly increased.

LysoPC-induced apoptosis was confirmed by TUNEL assay in human coronary artery SMCs treated with 30 or 60 μmol/L lysoPC for 24 h (Figure 2D). The cells with TUNEL-positive nuclei (green) were significantly increased by 30 and 60 μmol/L lysoPC (16.0±2.2% and 25.3± 4.3% *vs*. 5.07±1.7% of vehicle control (*n* = 3, *P*< 0.01) (Figure 2E). In addition, caspase-3 activity was determined in cells treated with 30 or 60 μmol/L lysoPC for 24h. The ratio of caspase-3 activity was increased to 1.3 ± 0.1 and 1.9 ± 0.1 of vehicle control respectively by 30 μmol/L and 60 μmol/L lysoPC (*n* = 3, *P* < 0.05 or *P* < 0.01) (Figure 2F). These results indicate that the reduced viability by lysoPC is related to induction of apoptosis in human coronary artery SMCs.

### Effect of lysoPC on Ca^2+^_i_

A previous study demonstrated that lysoPC induced a sustained Ca^2+^_i_ increase in human coronary artery SMCs, and the potential mediation of TRP channels was proposed [12]; however, the involvement of specific TRP channels was not understood. To specify which TRP channels mediate the lysoPC-induced Ca^2+^_i_ increase and apoptosis, we determined the effect of lysoPC on cytosolic Ca^2+^ by a confocal laser scanning technique in human coronary artery SMCs with different pharmacological tools.

**Figure 3:**
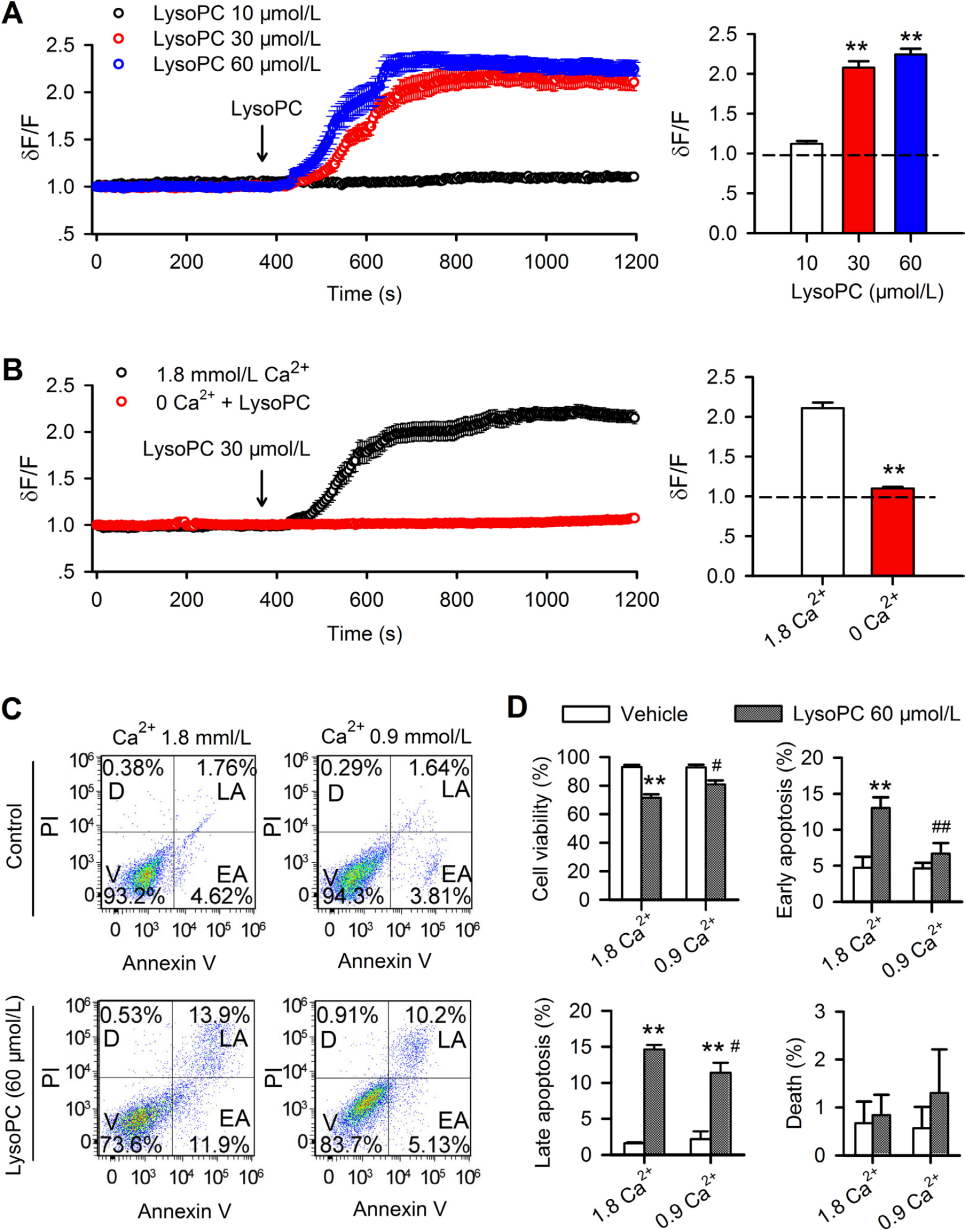
Effect of lysoPC on cytosolic Ca^2+^ **A.** Cytosolic free Ca^2+^ was determined in Tyrode's solution containing 5% FBS with a confocal microscopy technique in human coronary artery SMCs preloaded with Fluo-3 AM (data are mean ± SEM). The pseudoratio dF/F was applied to express intracellular free Ca^2+^ level, where dF is the measured fluorescence intensity of fluo 3, and F is the initial level of fluorescence intensity. LysoPC 30 μmol/L (*n* = 27) or 60 μmol/L (*n* = 27, **P < 0.01 *vs*. 10 μmol/L lysoPC), but not 10 μmol/L lysoPC (*n* = 25), induced a significant sustained Ca^2+^ influx. **B.** LysoPC (30 μmol/L) induced sustained Ca^2+^ increase (data are mean ± SEM) in the presence of 1.8 mmol/L Ca^2+^ in bath medium (*n* = 28), but not in the medium with 0 Ca^2+^ and 1 mmol/L EGTA (*n* = 26, **P < 0.01 *vs*. 1.8 mmol/L Ca^2+^). **C.** Cell apoptosis determined in 1.8 and 0.9 mm/L Ca^2+^ medium in human coronary artery SMCs by co-staining with PI and Annexin V-FITC (V, viability; D, death; LA, late apoptosis; EA, early apoptosis.) after treatment with 60 μmol/L lysoPC for 24 h. **D.** Percent values of viability, early apoptosis, late apoptosis and death after treatment with 60 μmol/L lysoPC. (*n* = 3, *P < 0.05, **P < 0.01 *vs*. vehicle control; #*P* < 0.05, ##*P* < 0.01 *vs*. 1.8 mmol/L Ca^2+^).

Application of 10 μmol/L lysoPC induced little change of Ca^2+^_i_ level, while 30 μmol/L or 60 μmol/L lysoPC induced a significant sustained Ca^2+^_i_ increase within 2 min in human coronary artery SMCs. Significant increase of relative Ca^2+^_i_ level at end of experiments was observed with 30 μmol/L or 60 μmol/L (Figure 3A, *n* = 26-28, *P* < 0.01 *vs*. 10 μmol/L). In contrast, omitting bath Ca^2+^ (Ca^2+^-free plus 1 mmol/L EGTA) abolished the sustained calcium increase induced by 30 μmol/L lysoPC (Figure 3C, *n* = 26-28, *P* < 0.01 *vs*. 1.8 mmol/L Ca^2+^), indicating that increase of Ca^2+^_i_ by lysoPC is related to Ca^2+^ influx.

To investigate the potential involvement of Ca^2+^ influx in lypoPC-induced apoptosis, the apoptosis was analyzed by flow cytometry in cells incubated in a culture medium with reducing Ca^2+^ from 1.8 mmol/L to 0.9 mmol/L. Figure 3C shows the cell viability, early apoptosis, late apoptosis and death in cells treated with lysoPC (60 μmol/L) treatment for 24 h. LysoPC-induced decrease of cell viability and increase of early and late apoptosis were reduced in culture medium containing 0.9 mmol/L Ca^2+^ (Figure 3D, *n* = 3, *P* < 0.05 or *P* < 0.01 *vs*. 1.8 mmol/L Ca^2+^). This suggests that lysoPC-induced apoptosis is related to the increase of Ca^2+^ influx by lysoPC.

### Effect of TRP channel blockade on apoptosis induced by lysoPC

**Figure 4:**
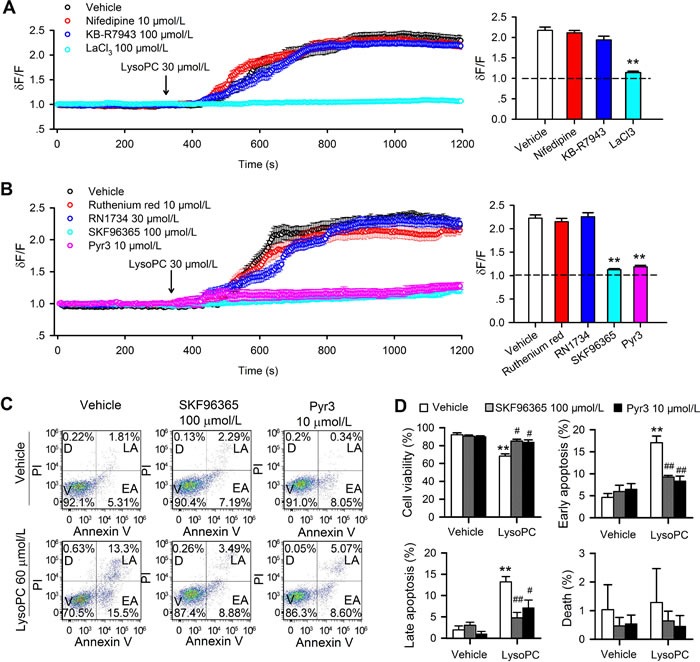
Effect of TRPC channel blockers on lysoPC-induced Ca^2+^ influx and apoptosis **A.** Ca^2+^ influx induced by 30 μmol/L lysoPC in cells pretreated with vehicle (*n* = 26), 10 μmol/L nifedipine (*n* = 26), 100 μmol/L KB-R7943 (*n* = 26) or 100 μmol/L LaCl_3_ (*n* = 26, **P < 0.01 *vs*. vehicle) **B.** Ca^2+^ influx induced by lysoPC in cells pretreated with vehicle (*n* = 27), 10 μmol/L Ruthenium Red (*n* = 27), 30 μmol/L RN1734 (*n* = 27), 100 μmol/L SKF-96365 (*n* = 27, **P < 0.01 *vs*. vehicle) or 10 μmol/L Pyr3 (*n* = 27, **P < 0.01 *vs*. vehicle). **C.** Cell apoptosis was determined in cells treated with 60 μmol/L lysoPC in the absence (vehicle control) and presence of 100 μmol/L SKF-96356 or 10 μmol/L Pyr3. **D.** Percent values of cell viability in cells treated with 60 μmol/L lysoPC in the absence (vehicle control) and presence of 100 μmol/L SKF-96356 or 10 μmol/L Pyr3 (*n* = 3 individual experiments, **P < 0.01 *vs*. vehicle; #*P* < 0.05, ##*P* < 0.01 *vs*. lysoPC alone).

Earlier reports demonstrated that Ca^2+^ flux was mainly mediated by voltage-gated Ca^2+^ channels (VGCC), Na^+^-Ca^2+^ exchanger (NCX), and/or TRP channels [18–21]. To obtain information on which pathway mediates the Ca^2+^ influx induced by lysoPC the L-type Ca^2+^ channel blocker nifedipine, the NCX inhibitor KB-R7943 [22], and nonselective TRP channel blocker La^3+^ were employed (Figure 4A). Interestingly, lysoPC-induced Ca^2+^ influx was prevented in human coronary artery SMCs by LaCl_3_ (100 μmol/L, *n* = 26, *P* < 0.01 *vs*. vehicle), but not by nifedipine (10 μmol/L, *n* = 26) or KB-R7943 (100 μmol/L, *n* = 28). The ratio values of relative Ca^2+^ level at end of experiments are illustrated in right panel of Figure 4A. These results suggest that Ca^2+^ influx induced by lysoPC is through TRP channels, but not VGCC or NCX.

It is generally believed that TRPC and TRPV are the major subclasses of TRP channels that mediate Ca^2+^ influx [9]. The general TRPC channel blocker SKF-96365 [23], the specific TRPC3 blocker Pyr3 [24], the TRPV2 channel blocker ruthenium red [25, 26], and the TRPV4 blockers RN1734 [26] were therefore tested in human coronary artery SMCs. Figure 4B shows the effects of different TRP channel blockers on lysoPC-induced Ca^2+^ influx. LysoPC-induced Ca^2+^_i_ increase was not affected in cells pretreated with 10 μmol/L ruthenium red or 30 μmol/L RN1734, but was clearly reduced in cells pretreated with 100 μmol/L SKF-96365 or 10 μmol/L Pyr3. The ratio values of relative Ca^2+^ levels at end of experiments are shown in the right panel of Figure 4B (*n* = 27-30, *P* < 0.01 *vs*. vehicle control). These results indicate that TRPC channels, but not TRPV2 or TRPV4 channels, are involved in lysoPC-induced Ca^2+^ influx in human coronary artery SMCs.

To determine whether blockade of TRPC channels would antagonize the apoptosis induced by lysoPC, cell viability (Figure S1A) was determined with MTT method in cells treated with lysoPC in the presence of TRPC channel blockers SKF96365 and Pyr3 (Figure S1A). SKF-96365 (100 μmol/L) and Pyr3 (10 μmol/L) significantly countered the increase of apoptosis and the decrease of cell viability induced by 30 or 60 μmol/L lysoPC (*n* = 3, *P* < 0.05 *vs*. lysoPC alone). Flow cytometry was conducted to analyze human coronary artery SMCs treated with 60 μmol/L lysoPC in the absence and presence of 100 μmol/L SKF-96365 or 10 μmol/L Pyr3 (24 h incubation) (Figure 4C). Figure 4D illustrates the mean percentage values of cell viability, early apoptosis, late apoptosis, and death. Pretreatment with 100 μmol/L SKF-96365 or 10 μmol/L Pyr3 significantly rescued lysoPC-induced decrease of cell viability and increase of early and late apoptosis (*n* = 3, *P* < 0.05 or *P* < 0.01 *vs*. vehicle). These results suggest that lysoPC-induced apoptosis is related to the activation of TRPC channels and Ca^2+^ influx.

### Effect of TRP channel blockade on apoptosis induced by lysoPC

**Figure 5:**
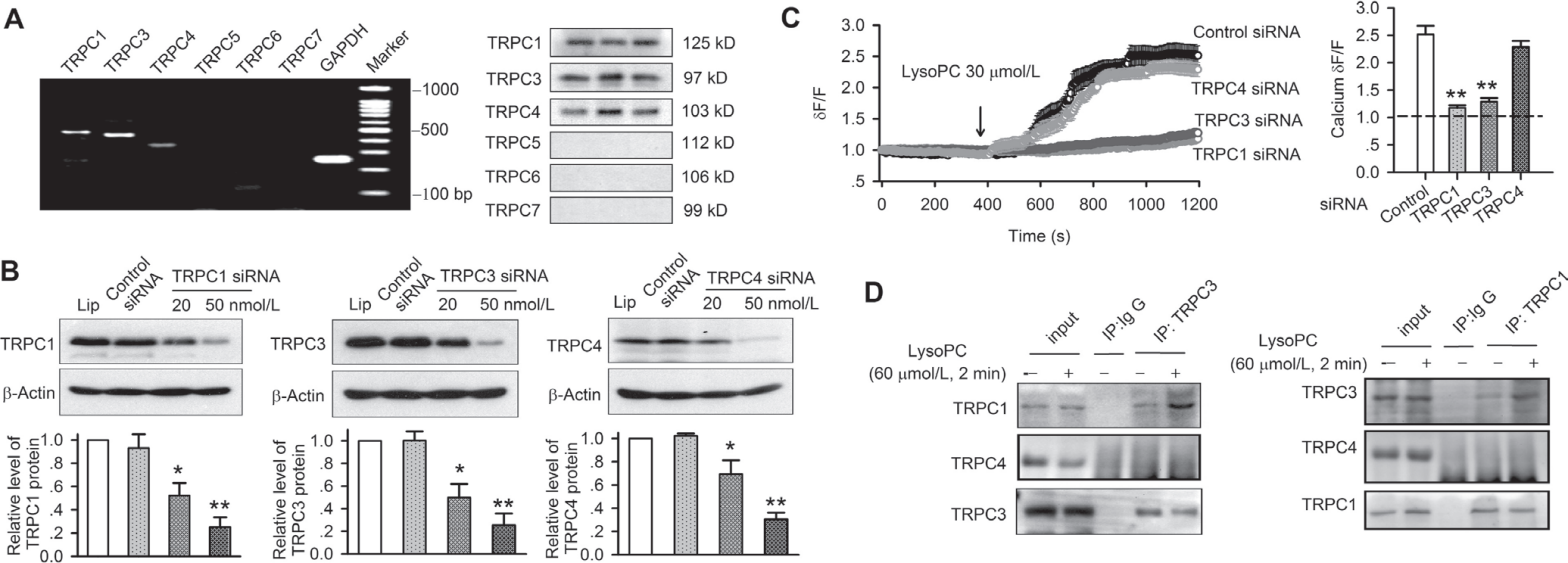
Silencing TRPC1, TRPC3 or TRPC4 channels and TRPC protein-protein interaction **A.** RT-PCR (left) and Western blots (right) of TRPC isoforms in human coronary artery SMCs (passages 3, 4, and 6). **B.** Western blots and ratio of protein levels of TRPC1, TRPC3 or TRPC4 channels in human coronary artery SMCs transfected with 20 or 50 nmol/L siRNAs targeting TRPC1, TRPC3 or TRPC4 channels (relative to b-actin and lipofectamine 2000, *n* = 3 individual experiments, *P < 0.05, **P < 0.01 *vs*. control siRNA or lipofectamine). **C.** Ca^2+^ influx (data are mean ± SEM) induced by 30 μmol/L lysoPC in cells transfected with 50 nmol/L control siRNA (*n* = 27), TRPC1 siRNA (*n* = 27, **P < 0.01 *vs*. control siRNA), TRPC3 siRNA (*n* = 28, **P < 0.01 *vs*. control siRNA), or TRPC4 siRNA (*n* = 28). **D.** Co-immunoprecipitation showing the interaction between TRPC1 and TRPC3 proteins in cells treated without or with 60 μmol/L lysoPC. IP indicates the antibody used to pull down the interacting proteins. Ig G represents the negative control.

There are six tissue specific isoforms of TRPC channels in humans [16]. To demonstrate which specific isoforms are involved in lysoPC-induced Ca^2+^ influx and apoptosis, the gene expression of TRPC channels was initially determined with RT-PCR, and the channel proteins were then verified with Western blots in human coronary artery SMCs (Figure 5A). The genes and the proteins of TRPC1, TRPC3 and TRPC4 were evident, but not TRPC5, TRPC6, and TRPC7, in human coronary artery SMCs.

To investigate how these three types of TRPC channels mediate the lysoPC-induced Ca^2+^ influx and apoptosis in human coronary artery SMCs, siRNA molecules targeting TRPC1, TRPC3 or TRPC4 channels were transfected into human coronary artery SMCs. Figure 5B illustrates the Western blots of TRPC1, TRPC3 or TRPC4 channels in human coronary artery SMCs transfected with 20 or 50 nmol/L corresponding siRNAs. The relative protein levels of TRPC1, TRPC3 or TRPC4 were significantly reduced by silencing TRPC1, TRPC3 or TRPC4 gene (*n* = 3, *P* < 0.05 or *P* < 0.01 *vs*. control siRNA or lipofectamine 2000).

The effect of lysoPC on Ca^2+^_i_ was further examined in human coronary artery SMCs transfected with 50 nmol/L corresponding siRNA. Figure 5C shows that the significant Ca^2+^ influx was not observed in cells with silenced TRPC1 or TRPC3 channels, while significant Ca^2+^ influx was seen in cells transfected with control siRNA or TRPC4 siRNA, and the ratio values of Ca^2+^ levels at end experiments in cells with transfecting siRNA molecules are shown in the right panel of Figure 5C (*n* = 28, *P* < 0.01 *vs*. control siRNA). These results indicate that TRPC1 or TRPC3 channels, but not TRPC4 channels, are involved in lysoPC-induced Ca^2+^ influx. It should be noted that silencing either TRPC1 or TRPC3 diminished lysoPC-induced Ca^2+^ influx. This implies that the heterogeneous channel complex composed of both TRPC1 and TRPC3 subunits is likely involved in lysoPC-induced Ca^2+^ influx in human coronary artery SMCs.

### Interaction of TRPC1 and TRPC3

The protein-protein interaction of TRPC1 and TRPC3 was determined by co-immunoprecipitation. Anti-TRPC1 or anti-TRPC3 was used to pull down TRPC1, TRPC3, or TRPC4 protein (Figure 5D). Co-immunoprecipitation revealed that the protein bands for TRPC1 and TRPC3 were observed at the expected molecular weight. The interaction protein bands of TRPC1 and TRPC3 were increased by lysoPC treatment (60 μmol/L for 2 min). No TRPC4 interaction bands were precipitated with TRPC1 or TRPC3. These results indicate that TRPC1 and TRPC3 subunits are interacted with each other to form TRPC1/TRPC3 channel complex, and mediate lysoPC-induced Ca^2+^ influx.

### Effects of silencing TRPC channels on cell viability and apoptosis induced by lysoPC

LysoPC-induced apoptosis was further analyzed in human coronary artery SMCs with silencing TRPC1, TRPC3 or TRPC4. Cell viability was determined in human coronary artery SMCs transfected with 50 nmol/L corresponding siRNA. The viability (Figure S1B) was decreased in cells treated with 30 or 60 μmol/L lysoPC (*n* = 3, *P* < 0.01 *vs*. control). LysoPC-induced reduction of cell viability was attenuated in cells with transfecting TRPC1 siRNA or TRPC3 siRNA (*n* = 3, *P* < 0.05 *vs*. control siRNA), but not TRPC4 siRNA. These results support the notion that TRPC1/TRPC3 channels, but not TRPC4, is involved in lysoPC-induced reduction of cell viability and apoptosis.

**Figure 6:**
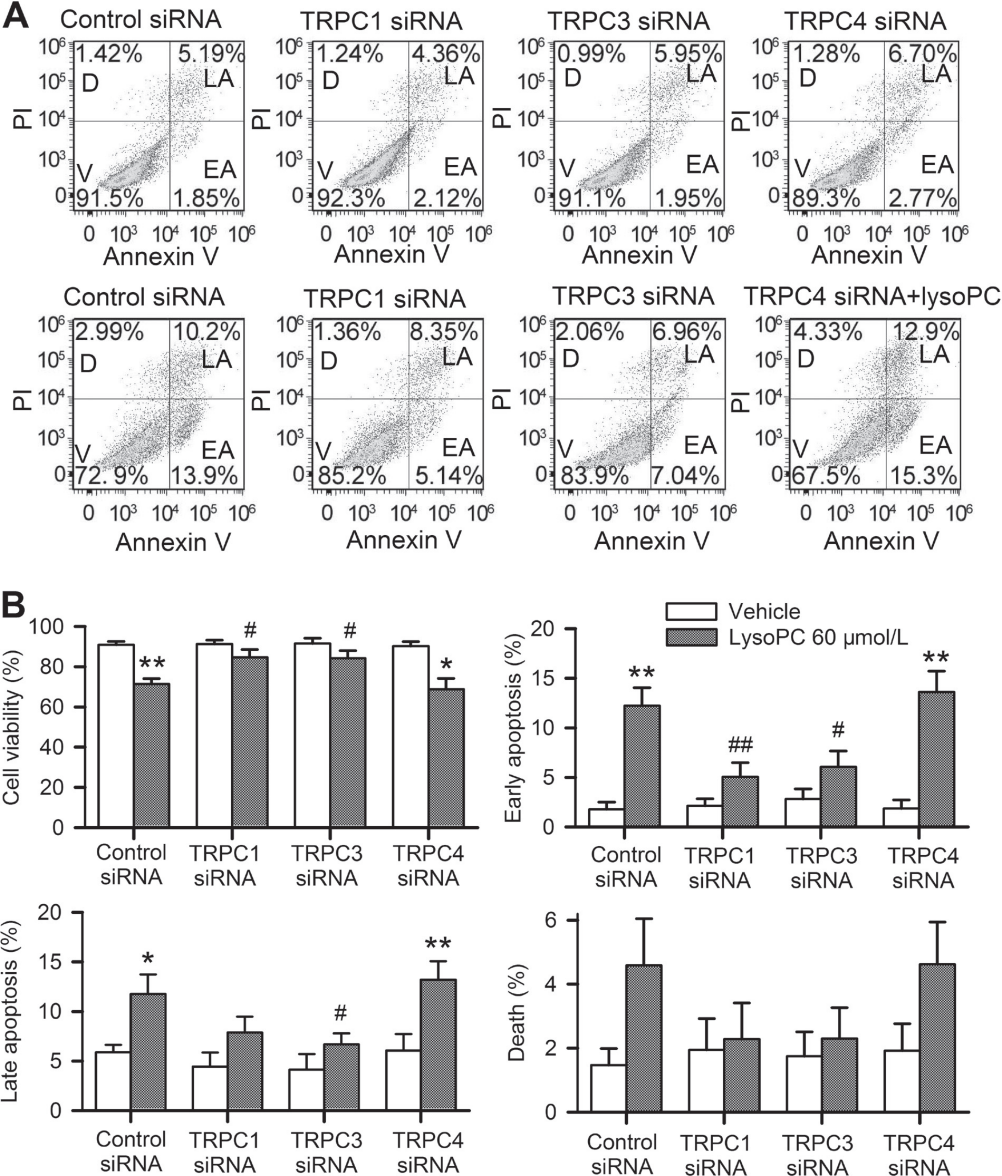
Effects of silencing TRPC channels on cell apoptosis **A.** Flow cytometry analysis in cells transfected with 50 nM control siRNA, TRPC1 siRNA, TRPC3 siRNA or TRPC4 siRNA for 72 h and stained with PI and Annexin V-FITC after incubating with vehicle (upper panel) or 60 μmol/L lysoPC (lower panel) for 24 h (V, viability; D, death; LA, late apoptosis; EA, early apoptosis). **B.** Percentage of cells showing viability, early apoptosis, late apoptosis, and death analyzed by flow cytometry. Data are mean ± SEM (*n* = 3, *P < 0.05, **P < 0.01 *vs*. vehicle, #*P* < 0.05, ##*P* < 0.01 *vs*. control siRNA with lysoPC).

Then, a higher concentration of 60 μmol/L lysopC was chosen to treat the cells to obtain the significant changes in 24 h incubation for flow cytometry analysis. Figure 6A shows the original graphs of apoptosis analysis with flow cytometry. Silencing TRPC1 or TRPC3, but not TRPC4, preserved cell viability in cells treated with 60 μmol/L lysoPC. Figure 6B illustrates the mean percentage values of cells that show viability, early apoptosis, late apoptosis, and death. LysoPC could decrease cell viability, and increase early and late apoptosis in cells transfected with control siRNA or TRPC4 siRNA (*n* = 3, *P* < 0.05 or *P* < 0.01 *vs*. control siRNA), but not in cells transfected with TRPC1 siRNA or TRPC3 siRNA.

### Molecular signals involved in lysoPC-induced apoptosis

**Figure 7:**
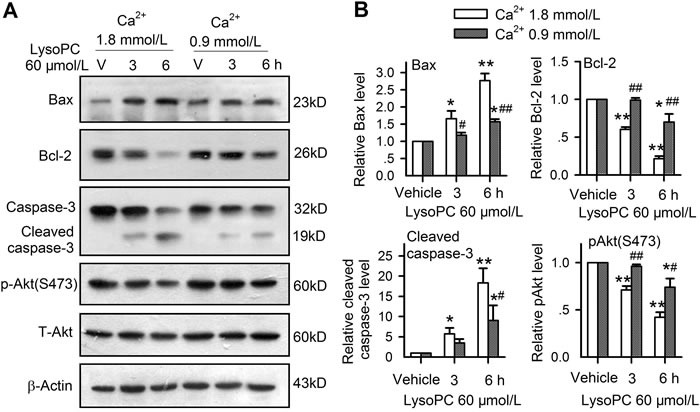
Intracellular signaling involved in apoptosis induced by lysoPC **A.** Western blots of Bax, Bcl-2, caspase-3, and p-Akt(S473) in human coronary artery SMCs treated with 60 μmol/L lysoPC for 3 h or 6 h incubation with medium containing 1.8 or 0.9 mmol/L Ca^2+^. **B.** Relative ratio of Bax, Bcl-2, caspase-3, or p-Akt (*n* = 3, *P < 0.05, **P < 0.01 *vs*. vehicle; #*P* < 0.05, ##*P* < 0.01 *vs*.1.8 mmol/L Ca^2+^).

Considered the importance of Ca^2+^ in lysoPC-induced apoptosis, the effects of lysoPC on apoptosis-related proteins were determined in cells cultured with 1.8 and 0.9 mmol/L and treated with 60 μmol/L lysoPC for 3 and 6 h. It was found that the pro-apoptotic proteins Bax and cleaved caspase-3 were increased, whereas anti-apoptotic protein Bcl-2 and the survival kinase p-Akt were reduced by lysoPC at 1.8 mmol/L Ca^2+^, and the effects were attenuated at 0.9 mmol/L extracellular Ca^2+^ (Figure 7A). Figure 7B illustrates the relative levels of Bax, cleaved caspase-3, Bcl-2 and p-Akt. LysoPC 60 μmol/L incubation with 1.8 mmol/L Ca^2+^ significantly increased the pro-apoptotic proteins Bax and cleaved caspase-3 (*n* = 3, *P* < 0.05 of *P* < 0.01 *vs*. vehicle) and decreased the anti-apoptotic protein Bcl-2 and the survival kinase p-Akt (*n* = 3, *P* < 0.05 or *P* < 0.01 *vs*. vehicle), and the effects were attenuated in cells incubated with 0.9 mmol/L Ca^2+^. Similar effects were observed for changes in apoptotic genes, which is determined using real time PCR in cells incubated with 1.8 or 0.9 mmol/L Ca^2+^ (Figure S2A). These results suggest that lysoPC-induced apoptosis is related to the activation of pro-apoptotic kinases and the inhibition of anti-apoptotic and survival kinases, which is correlated to bath Ca^2+^ concentration.

**Figure 8:**
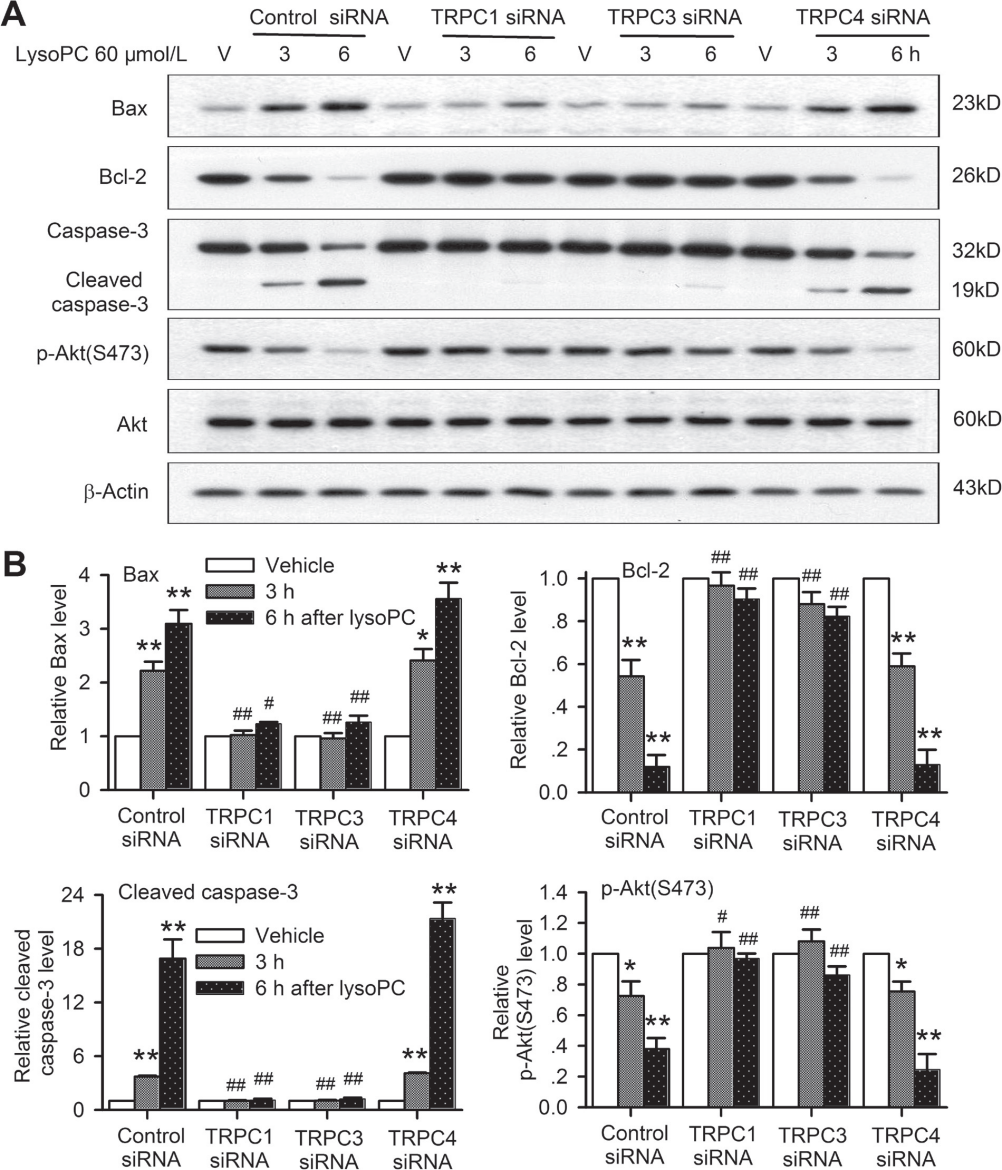
TRPC channels are involved in apoptotic signaling induced by lysoPC **A.** Western blots of Bax, Bcl-2, caspase-3, and p-Akt in human coronary artery SMCs transfected with 50 nM control siRNA, TRPC1 siRNA, TRPC3 siRNA or TRPC4 siRNA for 72 h, and then treated with vehicle (V) or 60 μmol/L lysoPC for 3 and 6 h. **B.** Relative mean levels of Bax, Bcl-2, caspase-3, and p-Akt in human coronary artery SMCs with the same treatment as in A (*n* = 3, *P < 0.05, **P < 0.01 *vs*. vehicle, #*P* < 0.05, ##*P* < 0.01 *vs*. control siRNA).

These molecular signals involved in lysoPC-induced apoptosis were then determined in human coronary artery SMCs with silenced TRPC channels. It is interesting to note that 60 μmol/L lysoPC increased Bax and cleaved caspase-3, and decreased Bcl-2 and p-Akt in cells transfected with control siRNA or TRPC4 siRNA, but not with TRPC1 or TRPC3 (Figure 8A). Figure 8B illustrates the mean relative levels of Bax, cleaved caspase-3, Bcl-2 and p-Akt. Similar changes in the apoptotic genes detected using real time PCR (Figure S2B) were observed in human coronary artery SMCs transfected with TRPC1 siRNA or TRPC3 siRNA molecules. Silencing TRPC1 or TRPC3 significantly prevented the lysoPC-induced increase of the pro-apoptotic proteins/genes Bax and caspase-3 and decrease of the anti-apoptotic protein/gene Bcl-2 and the survival kinase p-Akt in human coronary artery SMCs.

## DISCUSSION

It is generally recognized that lysoPC derived from cell membrane phosphatidylcholine is an important cell signaling molecule [27]. It is also a major phospholipid component of ox-LDL as a bioactive pro-inflammatory lipid generated by pathological activities. LysoPC exists in the blood plasma [28] at 140 and 150 μmol/L in free or bound form (with albumin) [29]. It has been reported that lysoPC is 5-fold higher in ox-LDL than in normal LDL [6]. LysoPC is therefore implicated as a critical factor in atherogenesis of ox-LDL [3, 4], and plays an important role in inflammatory disorders by altering various functions in a number of cell-types, including endothelial cells, SMCs, monocytes, macrophages, and T-cells [27]. LysoPC can be detected frequently in atherosclerotic lesions [30]. Although there is no doubt that lysoPC plays a role in atherogenesis, the detailed molecular mechanisms are not fully understood. An earlier study demonstrated that lysoPC increases Ca^2+^_i_ via stimulating platelet-activating factor receptor in mouse macrophages, which correlates to the pathogenesis of atherosclerosis [31].

Actually, lysoPC-induced Ca^2+^ influx was described in cardiac myocytes early in two decades ago [32, 33]. However, the potential pathways of lysoPC-induced Ca^2+^ influx are not fully understood. For instance, the reports whether L-type Ca^2+^ channel (I_Ca.L_) is involved in Ca^2+^ influx by lysoPC are controversial. Inhibition of I_Ca.L_ by lysoPC was reported in guinea pig atrial [34] and ventricular myocytes [32], and increase of I_Ca.L_ by lysoPC was observed in rat ventricular myocytes [35] and cultured rabbit portal vein smooth muscle cells [36]. On the other hand, L-type Ca^2+^ channel blocker verapamil significantly inhibited lysoPC-induced Ca^2+^ influx in *Drosophila* S2 cells [37], a very slight inhibition was observed in cultured coronary artery SMCs [13] and no inhibition was seen in check ventricular myocytes [33]. The discrepancy among these results may be related to differential types of cells and/or experimental conditions. The present observation and the previous report [38] support the notion that L-type Ca^2+^ channel is not involved in lysoPC-induced Ca^2+^ influx in human coronary artery SMCs, because the Ca^2+^ influx was not affected by the L-type Ca^2+^ channel blocker nifedipine.

Recent studies showed that lysoPC-induced increase of Ca^2+^_i_ is probably resulted from activation of TRP channels in human [38] and rabbit [13] coronary artery SMCs and in human endothelial cells [14, 39], which may participate in the atherogenesis [5–7]. However, it is not clear that the specific isoform(s) of TRP channels are involved. The present study demonstrates that TRPC1 and TRPC3 isoforms interact with each other to co-assembly form the heterogeneous channel complex in human coronary artery SMCs and mediate lysoPC-induced Ca^2+^ influx, thereby cause apoptosis via activating the pro-apoptotic proteins Bax and cleaved caspase-3 and inhibiting the anti-apoptotic protein Bcl-2 and the survival kinase p-Akt. Silencing TRPC1 or TRPC3 isoform diminishes the Ca^2+^ influx and inhibits the lysoPC-induced apoptosis.

It has been well documented that cytosolic Ca^2+^ is a key regulator of cellular biology, physiology and pathophysiology [9]. Cellular Ca^2+^ overload is highly toxic, and severe Ca^2+^ dysregulation can promote cell death directly through necrosis, while a more controlled cytosolic Ca^2+^ increase induced by milder insults promotes cell death through apoptosis [40]. The present study demonstrated that TRPC1/TRPC3 channels mediate lysoPC-induced Ca^2+^ influx and decrease cell viability. The reduction of cell viability by lysoPC is mainly resulted from apoptosis, suggesting that Ca^2+^ influx by lysoPC via activating TRPC1/TRPC3 channels is a milder insult in human coronary artery SMCs. Therefore, although Ca^2+^ influx induced by lysoPC was observed in seconds to minutes, a 24 h incubation was required for significant effect on cell viability and apoptosis.

It is recognized that apoptosis of vascular SMCs plays an important role in generating human vascular disorders including restenosis after angioplasty, low levels of atherosclerotic plaques and vessel remodeling [41–43]. Ca^2+^ overloading is critically responsible for the apoptosis, and blockade of Ca^2+^ influx is an effective way to prevent atherogenesis [44, 45]. Ca^2+^-induced apoptosis is usually involved in Bcl-2 family proteins [46]. LysoPC-induced apoptosis was found mainly at early and late stages of apoptotic pathway, which is consistent with earlier reports that increase in cytosolic Ca^2+^ occurs at early and late stages of the apoptotic pathway [47, 48]. In this study, we also observed that lysoPC significantly decreased the anti-apoptotic protein Bcl-2 and increased the pro-apoptotic proteins Bax and cleaved caspase-3 in human coronary artery SMCs upon the Ca^2+^ influx. In addition, lysoPC-induced p-Akt reduction was also involved in vascular apoptosis by increasing Ca^2+^ influx via activating TRPC1/TRPC3 channels. This is consistent with a recent report in which Akt1 activation inhibits vascular SMC apoptosis during atherogenesis [43].

In the hearts, it is believed that TRPC channels mediate pathologic cardiac hypertrophy via increasing Ca^2+^ influx [49]. TRPC1/TRPC4 channel complex- dependent Ca^2+^ cycling was observed in adult cardiomyocytes. A background Ca^2+^ entry pathway mediated by TRPC1/TRPC4 channels is critical for development of pathological cardiac remodeling [50]. The present study demonstrated that lysoPC-induced Ca^2+^ influx was related to activation of TRPC1/TRPC3 channels; thereafter apoptosis process was initiated in cultured human coronary artery SMCs. Three lines of evidence support the conclusion that TRPC1 and TRPC3 together form Ca^2+^ entry channels and mediate apoptosis induced by lysoPC in this study. First, the general TRPC channel blocker SKF-96365 or the TRPC3 inhibitor Pyr3 decreased the Ca^2+^ influx and the reduced cell viability induced by lysoPC. Second, silencing TRPC1 or TRPC3 isoform abolished lysoPC-induced Ca^2+^ influx and apoptosis. Third, lysoPC-induced increase of the pro-apoptotic proteins Bax and cleaved caspase-3 and decrease of the anti-apoptotic protein Bcl-2 and the survival kinase p-Akt were not observed in cells with silencing TRPC1 or TRPC3.

Collectively, the present study provides the novel information that lypoPC induces Ca^2+^ influx as well as apoptosis by activating TRPC1/TRPC3 channels in human coronary artery SMCs, which may be involved in atherogenesis in humans. The future effort is required to investigate whether selective TRPC1/TRPC3 channel blockers can be clinically used for treating atherosclerosis in patients with high ox-LDL.

## METERIALS AND METHODS

### Human coronary artery SMCs

The primary human coronary artery SMCs were purchased from ScienCell Research Laboratories (Carlsbad, CA, USA) and cultured in α-MEM/F12 (Invitrogen, Hong Kong, China). The media were supplemented with 10% fetal bovine serum, 100 IU penicillin and 100 μg/mL streptomycin (Invitrogen). The cells from 3-8 passages were used for the experiments.

### Reagents and antibodies

LysoPC (#L1381), 3-(4,5-dimethylthiazol-2-yl)-2,5-diphenyltetrazolium bromide (MTT, #M2128), nifedipine (#7634), KB-R7943 (#K4144), LaCl3 (#449830), ruthenium red (#R2751), RN1734 (#0658), SKF96365 (#S7809) and Pyr3 (#P0032) were obtained from Sigma-Aldrich (St. Louis, MO, USA). The primary antibodies anti-TRPC1 (sc-133076), anti-TRPC3 (sc-514670), anti-TRPC4 (sc-15063), anti-TRPC5 (sc-18737), anti-Bax (sc-6236), and anti-Bcl-2 (sc-7382) were purchased from Santa Cruz Biotechnology (Santa Cruz, CA, USA). Anti-TRPC6 (BA3394), anti-TRPC7 (PB0272) were purchased from Boster Biotechnology (Wuhan, China). Anti-caspase-3 (#9662), anti-Akt (#9272) and anti-pAkt(S473) (#4060) were from Cell Signaling Technology (Danvers, MA, USA). S-MEM without Ca^2+^ medium (#11380037) were obtained from Gibco. Other reagents were described as in specified.

### Cell viability assay

The cell viability was determined with MTT assay as described previously [51, 52]. Briefly, human coronary artery SMCs were seeded in 96-well plates (Thermo Fisher Scientific, MA, USA) for 24 h and incubated with medium containing 5% FBS and different concentrations of lypoPC or equal volume of vehicle (ethanol, Sigma-Aldrich) for different period of time. The cells were then incubated with 0.5 mg/mL MTT for 3 h, and re-suspended in 150 μL of DMSO. Absorbance was measured at 575 nm using FLUOstar Omega (BMG Labtech's, Germany). The cells treated with DMSO were considered to be 100% viable.

### Flow cytometry analysis

Flow cytometric analysis [51, 52] was used to determine cell cycling progression and apoptosis. The cell cycling progression was determined in human coronary artery SMCs incubated with medium containing 5% FBS lypoPC at 30 or 60 μmol/L for 24 h. The cells were harvested and fixed in 70% cold methanol overnight at 4°C and then treated with RNase A and exposed to propidium iodide (PI) (Invitrogen) for 30 min at room temperature. The cell cycle data of flow cytometry were analyzed by FlowJo software (Ashland, OR, USA).

The apoptosis assay was made in human coronary artery SMCs harvested by EDTA-free trypsin. The cells were washed with PBS and then incubated at 4°C in a binding buffer (100 μL) containing 5 mg of propidium iodide and 5 μL of Annexin V-FITC (R&D Systems, Minneapolis, MN, USA) in the dark for 15 min. Afterwards, 900 μL binding buffer was added and fluorescence of Annexin V-FITC and propidium iodide were detected by Gallios Flow Cytometer (Beckman, USA) and data were analyze­­d with FlowJo software. Cells positive for Annexin V-FITC were considered to be apoptotic whereas cells positive for PI and negative for FITC-Annexin V were to be considered death.

### TUNEL assay

The TUNEL (terminal deoxynucleotidyl transferase dUTP nick end labeling kit from Beyotime Institute of Biotechnology (Shanghai, China) was employed to detect DNA fragmentation resulted from apoptosis following manufacturer's instruction. The human coronary artery SMCs were seeded on the coverslips and incubated in the culture medium containing 5% FBS lypoPC (30 or 60 μmol/L) for 24 h. The cells were fixed with 4% paraformaldehyde for 20 min after incubation of PBS with 0.2% Triton X-100 for 5 min. The cells were then treated with the mixture of terminal deoxynucleotidyl transferase enzyme, terminal deoxynucleotidyl transferase reaction buffer and fluorescent labeling buffer (1:24:25) at 37°C for 60 min. DAPI staining were used to count total cell number and the apoptotic cells with green nuclei. The apoptotic cells were quantitated by counting the number of TUNEL-positive cells in four microscopic fields randomly.

### Caspase-3 activity assay

Caspase-3 activity kit from Beyotime Institute of Biotechnology was used following manufacturer's instructions. Briefly, human coronary artery SMCs with different treatments were harvested, rinsed with PBS, re-suspended in lysis buffer, and then incubated on ice for 15 min. The lysate was centrifuged at 16,000 g at 4°C for 15 min. Approximately 70 μL of reaction buffer and 10 μL of caspase-3 substrate were mixed with 20 μL lysate supernatant, and then incubated in 96-well plates at 37°C for 4 h. Absorbance was measured at 405 nm using FLUOstar Omega (BMG LABTECH Inc., Cary, NC, USA). The caspase-3 activity was described as a percentage of control.

### Ca^2+^_i_ measurement

Ca^2+^_i_ in human coronary artery SMCs was determined with a confocal laser scanning microscope (Olympus FV300, Tokyo, Japan) as described previously [53]. Briefly, the cells were cultured in a 35 mm dish (Thermo Fisher Scientific, MA, USA) for 48 h and then loaded with 5 μmol/L fluo-3 AM (Biotium, Hayward, CA, USA) for 30 min at 37°C, incubated in serum-free bath solution for 40 min in the dark, and then washed with bath solution. The bath solution contains (in mmol/L) NaCl 140, KCl 5.0, MgCl2 1.0, CaCl2 1.8, HEPES 10, glucose 10 (pH adjusted to 7.3 using NaOH), and 5% FBS. Fluorescence was excited by an argon laser at 488 nm and emission was detected at 525 nm. The Ca^2+^_i_ level was expressed as ratio of basal fluorescence intensity.

### Messenger RNA measurement

Messenger RNAs of TRPC channels were determined in human coronary artery SMCs with reverse-transcription polymerase chain reaction (RT-PCR) as described previously [54]. Briefly, total RNA from cells was extracted using TriZol (Invitrogen) according to the manufacturer's instructions, and cDNA was generated with Primescript RT reagent kit (Takara, Dalian, China) and used as a template for the amplification using our previously designed TRPC primers [51, 52]. Real-time PCR (Applied Biosystems StepOnePlus™) was used to determine apoptosis-related genes in human coronary artery SMCs treated with lysoPC using SYBR Green qPCR Kit (Thermo Fisher Scientific, MA, USA) with primers as described previously [55]. Alterations in relative gene expression were determined by theDDCTmethod.

### Western blotting analysis and co-immunoprecipitation

The membrane proteins were determined with Western immunoblotting analysis as described previously. Briefly, cells were lysed with modified RIPA buffer (50 mmol/L Tris-Cl, pH 8, 150 mmol/L NaCl, 1% Nonidet P-40 (NP-40), 0.5% sodium deoxycholate, 1% SDS) for 30 min at 4°C, cell lysates were then centrifuged at 12,000g for 15 min at 4°C. After transferring the supernatant to a fresh ice-cold tube, protein concentration was determined with Bio-Rad protein assay. Equal concentrations of proteins were mixed with SDS sample buffer and denatured at 95°C for 5 min. Samples were resolved with 8% SDS-page gel. Gels were then transferred onto nitrocellulose membrane paper, and membranes were blocked with 5% non-fat dried milk in TTBS (0.1% Tween-20) for 1 h. After blocking, the blots were incubated overnight at 4°C in corresponding primary antibodies (1:1000-2000). After washing with TTBS, the membranes were incubated with HRP-conjugated secondary antibodies (1:5,000) (Santa Cruz Biotech) at room temperature for 1 h. Membranes were washed again with TTBS then processed to develop x-ray film using an enhanced chemiluminescence detection system (ECL; GE Healthcare, Bio-Science AB, Uppsala, Sweden). The relative band intensities were measured by image analysis software Gel-Pro Analyzer.

Co-immunoprecipitation was used to determine protein-protein interaction as described previously [51, 52]. Briefly, the cultured human coronary artery SMCs were treated with or without lysoPC for 2 min before the harvest. Then, equal amounts of proteins were immunoprecipitated with 2 μg of antibody at 4°C overnight. Then, 20 μL of protein A/G agarose beads (Santa Cruz Biotech) was added and incubate for 2~4 h at 4°C. Immunoprecipitated proteins bound to the pelleted protein A/G beads were washed thoroughly in PBS, denatured in Laemmli sample buffer, electrophoresed and blotted. Proteins were detected by using anti-TRPC1 and anti-TRPC3 primary antibody, respectively. As a negative control, protein samples were mock-immunoprecipitated with pre-immuno-IgG and treated in the same way.

### RNA interference

Small interfering RNA (siRNA) molecules targeting human TRPC1 (sc-42664), TRPC3 (sc-42666), TRPC4 (sc-42668), and negative control (sc-37007) were purchased from Santa Cruz Biotech. These siRNAs are target-specific 19-25nt siRNA designed to knock down gene expression with the procedure described previously [56]. Human coronary artery SMCs at 60~70% confluence were transfected with siRNA molecules at 20 or 50 nmol/L using lipofectamine 2000 (Invitrogen). 72 h after transfection, cells were used for determining Ca^2+^ activity, related gene and protein expression, and cell proliferation and apoptosis assays.

### Statistical analysis

Data are expressed as mean ± SEM, and statistical differences between groups were further evaluated by Student's t-test two group comparison or two-way ANOVA for multiple groups. Differences of *P* < 0.05 were considered to be significant.

## SUPPLEMENTARY MATERIAL


