# Supplementary material for: TRPC1/TRPC3 channels mediate lysophosphatidylcholine-induced apoptosis in cultured human coronary artery smooth muscles cells
Source: Oncotarget. 2016 Jul 26;7(32):50937–51. doi: 10.18632/oncotarget.10853 (PMC5239449; doi:10.18632/oncotarget.10853)
Supplement: Supplementary file 1 [file oncotarget-07-50937-s001.pdf]

TRPC1/TRPC3 channels mediate lysophosphatidylcholine-induced apoptosis in cultured human coronary artery smooth muscles cells

Supplementary Material

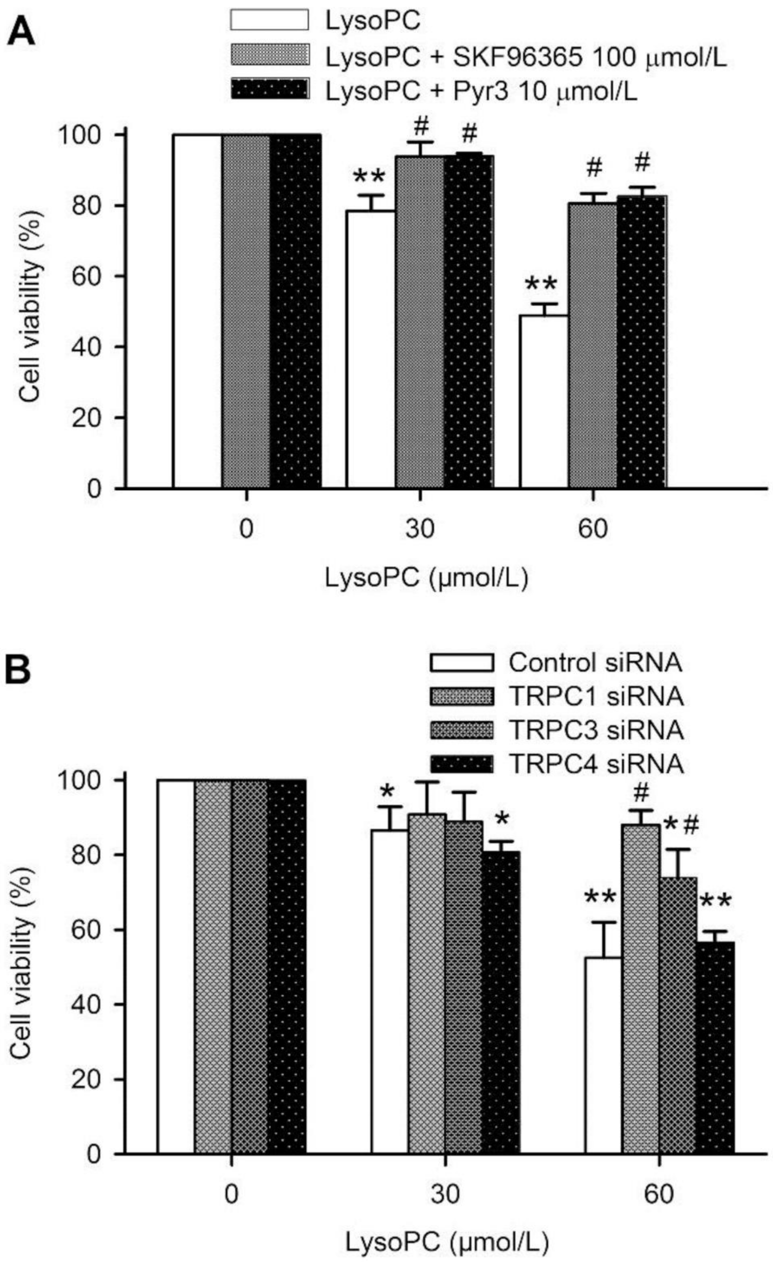

Figure S1. TRPC channels and cell viability induced by lysoPC. (A) Cell viability was determined with MTT in human coronary artery SMCs treated with 30 or 60 μmol/L lysoPC in the absence (vehicle control) and presence of 100 μmol/L SKF-96356 or 10 μmol/L Pyr3 (72 h incubation, n = 3 individual experiments, **P<0.01 vs. vehicle, #P<0.05 vs. lysoPC alone). **(B)** Cell viability changes induced by 30 and 60 μmol/L lysoPC (72 h) was determined in cells transfected with 50 nmol/L control siRNA, TRPC1 siRNA, TRPC3 siRNA or TRPC4 siRNA (n = 3 individual experiments, *P<0.05, **P<0.01 vs. vehicle, #P<0.05 vs. control siRNA).

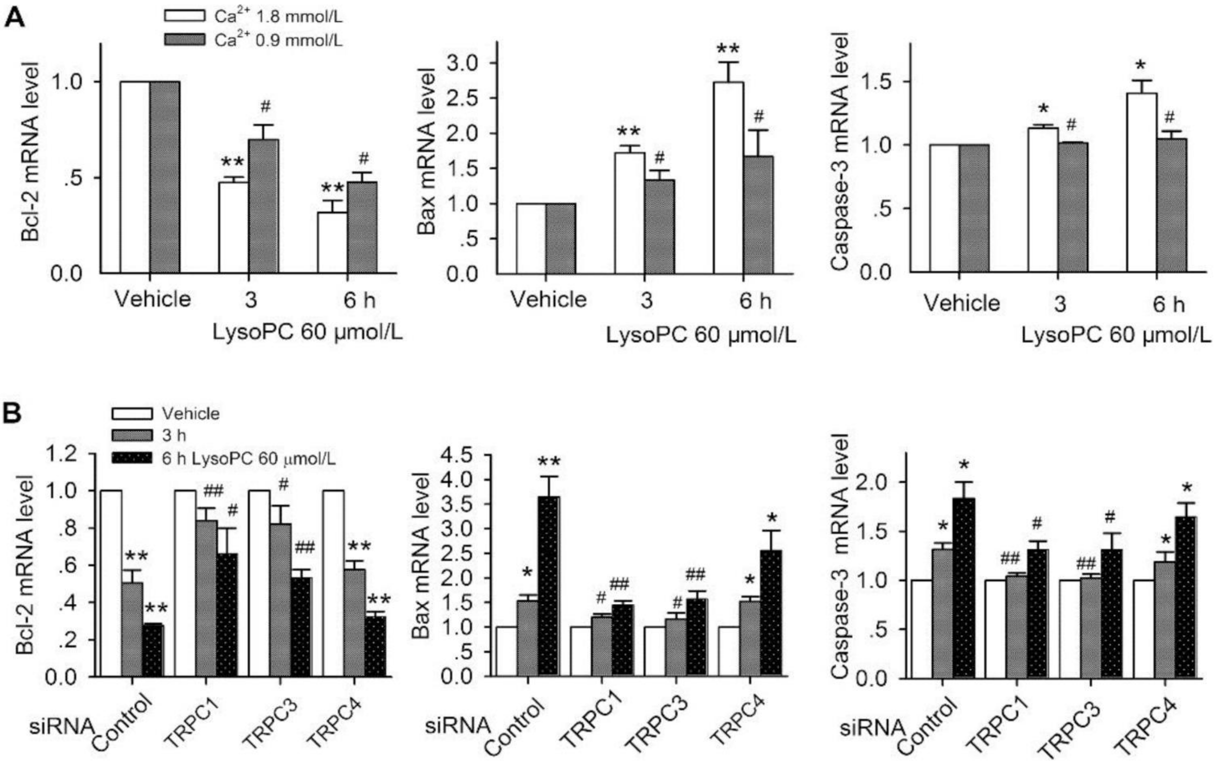

Figure S2. Changes of apoptosis-related genes induced by lysoPC. (A) Relative gene expression of Bcl-2, Bax and caspase-3 was determined using real-time PCR in human coronary artery SMCs treated with 60 μmol/L lysoPC for 3 h or 6 h under incubation with 1.8 mmol/L Ca^{2+} or 0.9 mmol/L Ca^{2+} (n = 3 experiments, *P<0.05, **P<0.01 vs. vehicle, #P<0.05 vs. Normal). **(B)** Relative gene expression of Bcl-2, Bax and caspase-3 in human coronary artery SMCs transfected with 50 nmol/L control siRNA, TRPC1 siRNA, TRPC3 siRNA or TRPC4 siRNA for 72 h, and then treated with vehicle or 60 μmol/L lysoPC for 3 and 6 h (n = 3 experiments, *P<0.05, **P<0.01 vs. vehicle, #P<0.05, ##P<0.01 vs. control siRNA).
